# Deep brain stimulation for dystonia treatment in cerebral palsy: efficacy exploration

**DOI:** 10.3389/ebm.2025.10330

**Published:** 2025-06-09

**Authors:** Haoyang Zheng, Duo Zhang, Wei Xiang, Yong Gan, Zesheng Peng, Yuyi Wu, Peng Fu

**Affiliations:** ^1^ Department of Neurosurgery, Union Hospital, Tongji Medical College, Huazhong University of Science and Technology, Wuhan, China; ^2^ Department of Nursing, Tongji Hospital, Tongji Medical College, Huazhong University of Science and Technology, Wuhan, Hubei, China; ^3^ Department of Social Medicine and Health Management, School of Public Health, Tongji Medical College, Huazhong University of Science and Technology, Wuhan, China

**Keywords:** dystonia, cerebral palsy, deep brain stimulation, functional neurosurgery, movement disorders

## Abstract

Dystonia, a challenging movement disorder, poses significant therapeutic challenges due to its resistance to treatment, resulting in both physical impairment and substantial mental distress, ultimately impacting overall quality of life. Cerebral palsy (CP) is a major non-genetic cause of secondary dystonia, characterized by diverse clinical presentations. This study aims to comprehensively evaluate the effectiveness of deep brain stimulation (DBS) as a therapeutic intervention for individuals with dystonic CP. We conducted a systematic analysis of studies assessing the safety and effectiveness of DBS, with a focus on its long-term outcomes [PROSPERO (Unique identifier: CRD42023399285)]. We examined factors that influence treatment response and proposed strategies to enhance patient quality of life. DBS, especially when targeting the basal ganglia or innovative targets, shows promise as a therapeutic approach for dystonic CP. While existing controlled studies confirm its safety and effectiveness, a thorough evaluation of long-term efficacy remains crucial. This research highlights the potential of DBS in improving the lives of individuals with dystonic CP, providing hope for further refinement, innovation, and broader clinical application of this therapeutic approach.

## Impact statement

This work is crucial to the field because dystonia, especially in the context of cerebral palsy (CP), presents significant therapeutic challenges due to its resistance to conventional treatments, leading to severe physical impairment and mental distress. Our study advances the field by providing a comprehensive evaluation of deep brain stimulation (DBS) as a therapeutic intervention for dystonic CP, emphasizing long-term outcomes. By systematically analyzing studies on the safety and effectiveness of DBS, and examining factors influencing treatment response, this research introduces new insights into optimizing DBS for better patient outcomes. The findings suggest that DBS, particularly targeting the basal ganglia or innovative targets, holds significant promise in improving the quality of life for individuals with dystonic CP. This new information could guide further refinement and broader clinical application of DBS, potentially revolutionizing treatment strategies for dystonic CP and offering new hope to affected individuals.

## Introduction

Dystonia is a movement disorder characterized by sustained or intermittent involuntary muscle contractions, which result in abnormal and repetitive movements or postures in affected individuals [1]. It is considered the third most common movement disorder, following Parkinson’s disease and essential tremor, with an estimated overall prevalence of 601.1 per million [2]. Typically, dystonia is triggered or worsened by voluntary actions and is associated with overflow muscle activation. This disorder can present a range of challenges, including pain, depressive symptoms, anxiety, social stigmatization, and reduced self-esteem, ultimately leading to a compromised health-related quality of life [3]. Depending on its underlying cause, dystonia can manifest as primary, hereditary, or idiopathic, or it can occur as a secondary consequence of various neurological or systemic disorders [4].

Dystonia can be divided into two main subtypes based on clinical manifestations: motor dystonia and postural dystonia. Motor dystonia is characterized by abnormal muscle tension during movement, resulting in involuntary twitches and tremors across various body regions. These movements can occur intermittently or continuously and often worsen during voluntary actions, affecting motor function and causing dyskinesia. Motor dystonia includes a wide range of movement patterns, from focal (affecting a specific body part) to generalized (involving multiple body regions). Examples of motor dystonia include writer’s cramp (focal hand dystonia), cervical dystonia (involving neck muscles), and generalized dystonia (affecting various body regions). Some forms of motor dystonia are specific to certain tasks, such as musician’s dystonia when playing a musical instrument. On the other hand, postural dystonia is characterized by atypical and sustained postures without the repetitive muscle movements seen in motor dystonia. These postures are often involuntary and can cause discomfort and pain. They typically remain stable over time and occur in specific postures. Common examples of postural dystonia are camptocormia (forward bending of the trunk), Pisa syndrome (lateral bending of the trunk), and anterocollis (forward flexion of the neck). These postures become more pronounced when the individual tries to stand or walk. Postural dystonia significantly affects an individual’s stability and balance, making it challenging to maintain an upright posture or engage in daily activities. In summary, both conditions have a significant impact on an individual’s quality of life, requiring management strategies that may include physical therapy, medication, and other interventions tailored to the specific dystonic symptoms.

Cerebral palsy (CP) is the most common condition associated with dystonia in children, often resulting from brain injury due to complications of prematurity, stroke, or hypoxic-ischemic encephalopathy [5]. The incidence of CP was 1.77 per 1,000 live births, and it was commonly accompanied by other conditions such as seizures, communication disorders, hearing and visual impairments, and intellectual disabilities [6]. Dystonia significantly affects various aspects of well-being in the CP population, including motor function, emotional balance, and Health-Related Quality of Life (HRQoL). Many patients experience severe physical disabilities due to abnormal movements, posturing, and musculoskeletal deformities [7].

CP is the most common non-genetic condition associated with secondary dystonia, and its clinical management presents significant challenges [8]. The main goals in treating dystonia associated with CP are to reduce dystonic symptoms, improve functional capacity, relieve pain, and enhance overall care [9]. Traditional approaches, such as medication and physical therapies, often produce unsatisfactory results. On the other hand, deep brain stimulation (DBS) has emerged as a key intervention for cases that do not respond to medical treatment [10]. DBS improves dystonic symptoms by electrically modulating abnormal neuronal signals in the basal ganglia’s output structure. This modulation has the potential to restore more effective signal transmission in the cortico-basal ganglia circuit, thereby reducing abnormal cortical excitability and normalizing synaptic plasticity in the motor cortex [11]. Recent studies have shown that DBS is significantly effective in myoclonus dystonia and tardive dystonia, which are types of primary dystonia [12]. However, the effectiveness of DBS in secondary dystonia is still being investigated and varies in different cases.

This review aims to provide a comprehensive evaluation of the impact of DBS surgery on the prognosis of functional adaptation in individuals with secondary dystonia associated with CP. In order to accomplish this, we have synthesized and analyzed the neurophysiological mechanisms, therapeutic effectiveness, factors influencing outcomes, and strategies for enhancing the results of DBS treatment in this particular group of patients.

## Neurophysiological mechanisms of DBS in dystonia management

The general concept of DBS is that high-frequency stimulation modulates abnormal neural activity and trains it into a pattern of normative behavior, with the possible mechanism being an inhibitory neuronal effect of the pulses on the somata. The inhibitory effect may manifest as a direct result of depolarization block, a phenomenon mediated by mechanisms that encompass sodium channel inactivation and augmentation of potassium currents. Furthermore, DBS may also increase and normalize signal output in the stimulated area by activating local axons [13]. Therefore, a thorough understanding of the neurophysiological basis of dystonia is essential for the development of DBS and new therapeutic approaches.

CP results from damage to the developing brain during pregnancy, birth, or early childhood. This damage affects important brain regions responsible for motor control, including the motor cortex, basal ganglia, cerebellum, and thalamus. Specific anatomic lesions are often found in CP, including diffuse cortical dysplasia and atrophic lobar sclerosis. These lesions can be observed as scattered scar-like marks resembling marbling within the cortex and basal nuclei. The regulation of normal muscle tone hinges on the dynamic balance between the inhibitory effect of descending cortical fibers and the facilitatory effect of peripheral afferent fibers. When brain injuries disrupt the descending cortical fiber tracts, this balance is disturbed. As a result, the inhibitory effect weakens, and the excitatory influence of peripheral afferent fibers becomes more prominent. Clinically, this disruption presents as spastic dyskinesia and postural anomalies.

In the past, dystonia was primarily believed to be caused by dysfunction in the basal ganglia. However, a new perspective suggests that other areas of the brain may also be involved. According to the emerging network model, dystonia can result from dysfunction in different parts of the brain network, dysfunction across multiple nodes, or abnormal communication between nodes [14]. The current evidence supports the idea that dystonia is a circuit disorder that affects the basal ganglia-thalamo-cortical and cerebello-thalamo-cortical pathways. This includes various regions such as the deep cerebellar nuclei, cerebellar cortex, pontine nuclei, basal ganglia, subthalamic nucleus, thalamus, and cerebral cortex [15].

The basal ganglia, a cluster of nuclei located deep within the brain, are widely acknowledged to play a pivotal role in the pathophysiology of dystonia arising from CP. In individuals with CP, damage to the basal ganglia leads to abnormal neuronal firing patterns and a compromised ability to inhibit unwanted movements. These nuclei are involved in various critical functions, including motor control, learning, cognition, motivation, emotion, and social behavior. The basal ganglia consist of the striatum, globus pallidus, subthalamic nucleus (STN), substantia nigra, and pedunculopontine nucleus (PPN). The cerebral cortex communicates with the basal ganglia through the corticostriatal pathway, which includes both the direct and indirect pathways [16]. The direct pathway projects from the striatum to the substantia nigra pars reticulata (SNr) and the globus pallidus interna (GPi), while the indirect pathway projects to SNr and GPi through the globus pallidus pars externa (GPe) and STN [17]. Additionally, there is growing evidence that cerebellar dysfunction may also contribute to the development of dystonia in CP. The cerebellum is involved in motor functions such as coordination, balance, and posture, as well as non-motor functions like language, social cognition, and emotion [18–21]. It has connections with the thalamus, vestibular nuclei, and inferior olive through monosynaptic projections. There are also polysynaptic short-latency connections between the cerebellum and key structures, including the basal ganglia [22]. This interconnectedness provides a basis for using DBS stimulation of the cerebellum in dystonia treatment, considering its anatomical and pathophysiological relationship with nuclei like the basal ganglia and thalamus.

Distinctions in the neurophysiological underpinnings of motor dystonia and postural dystonia are apparent. Motor dystonia primarily involves dysfunction within the basal ganglia, which plays a crucial role in motor regulation. Abnormalities in the basal ganglia can result in difficulties initiating and controlling voluntary movements. The connection between the basal ganglia and the cerebral cortex is essential for coordinating motor actions. The disrupted interaction between these regions is believed to contribute to the development of motor dystonia. On the other hand, postural dystonia focuses more on maintaining specific body positions rather than executing discrete movements. Postural dystonia often involves abnormalities in muscle tone regulation, leading to sustained muscle contractions and abnormal postures. In the case of postural dystonia, dysfunction of the direct pathway causes rigid hypertonia in childhood due to disinhibition of descending basal ganglia output [17]. Dysfunctions in the feedback loop between the sensory system, responsible for providing information about body position, and the motor system may have a stronger impact on postural dystonia. Although the cerebellum, located at the back of the brain, is not the primary area involved, abnormalities in this region can also contribute to postural dystonia. It is important to note that these are general descriptions, and there is a wide range of subtypes and variations within both motor and postural dystonia. Therefore, the specific causes and anatomical irregularities can vary considerably depending on the individual and the particular manifestation of dystonia they present.

The motor circuits involved in dystonia due to CP are highly complex and involve multiple regions within the brain. The basal ganglia, cerebellum, and cerebral cortex work together as an interconnected network, with the basal ganglia-to-cerebellum pathway playing a significant role in the manifestation of dystonic movements. Further research is needed to gain a better understanding of the underlying mechanisms and to develop effective therapeutic interventions for this condition.

## Efficacy of DBS in treating dystonia in CP

A comprehensive literature review was performed using the Web of Science and PubMed database to identify relevant English-language articles published until 1 June 2024. The retrieval details have been registered at PROSPERO (CRD42023399285). Search terms included cerebral palsy, secondary dystonia, and deep brain stimulation. Supplementary Material S1 provides detailed criteria and statistical methods. Each article was meticulously scrutinized for patient-centric information. The clinical outcomes were commonly evaluated using the Burke-Fahn-Marsden Dystonia Rating Scale Movement (BFMDRS-M) score [23].

**FIGURE 1:**
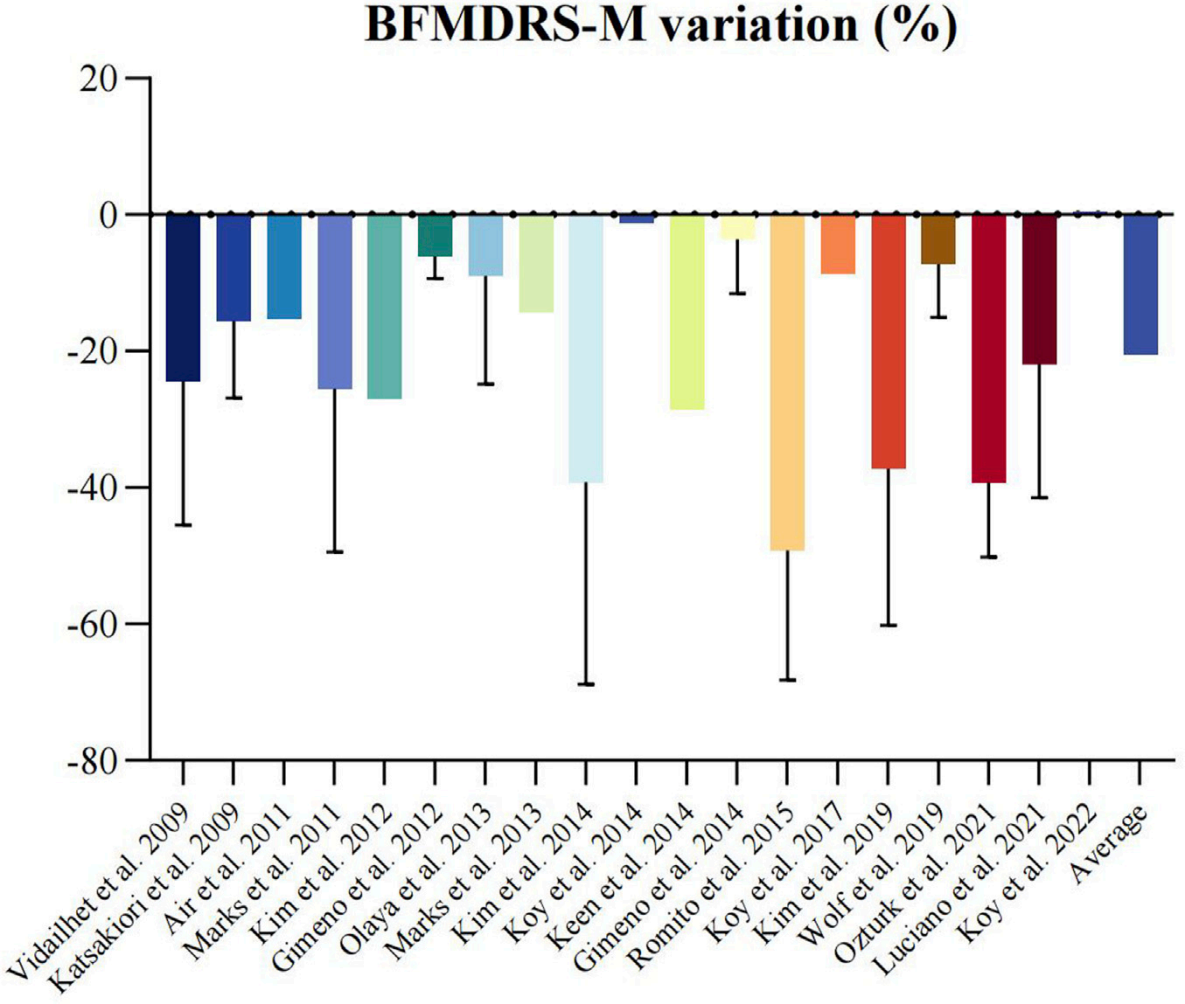
Forest plot of random effects meta-analysis of non-randomized studies reporting on dystonia before and after deep brain stimulation. Mean difference across studies reporting on BFMDRS-M scales.

**FIGURE 2:**
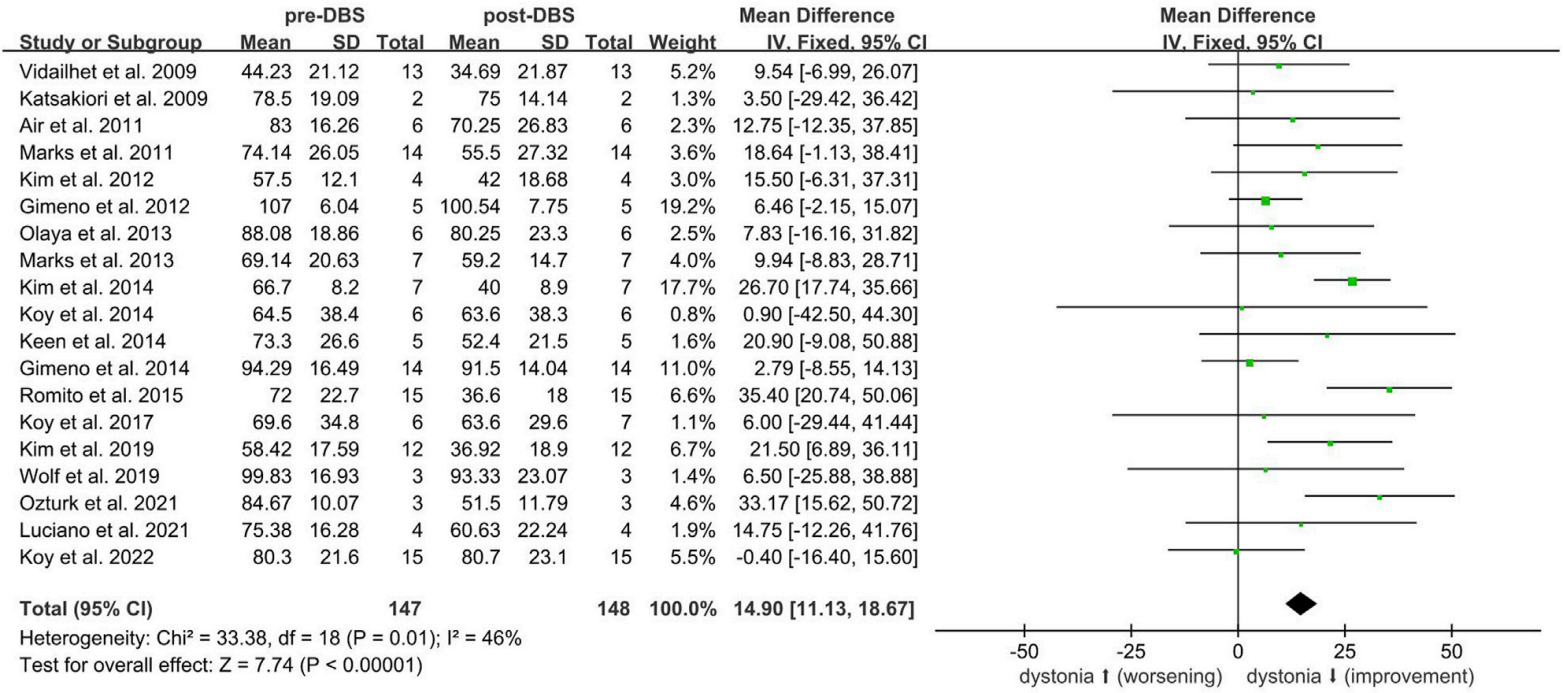
BFMRS-M scales variations in the individual studies on DBS in cerebral palsy. Negative values represent improvement. Standard error bars are showed.

Twenty-one non-randomized studies [7, 24–43] assessed the efficacy of DBS using the BFMDRS-M scores. A meta-analysis of 19 studies [7, 24–40, 43] (n = 148) showed that DBS significantly improved dystonia due to CP (SMD 14.90; 95% CI 11.13 to 18.67; Figure 1). The mean BFMDRS-M score was 73.74 ± 25.23 before the procedure and 60.10 ± 29.06 after the procedure, indicating an overall improvement of 20.46% (P < 0.001). Except for the study by Koy et al., all studies reported the effectiveness of DBS in reducing motor symptoms of dystonia. However, the degree of improvement varied considerably across the studies, with a range of 1.23%–49.22% reduction in the BFMDRS-M score (Figure 2). While the overall effect observed in the forest plot supports a beneficial role for DBS in CP-associated dystonia, the heterogeneity among studies was not negligible (Chi-square p = 0.01, I^2^ = 46%). Although an I^2^ value below 50% is often interpreted as low, in this context it may represent moderate heterogeneity. Potential sources of variability include differences in DBS implantation techniques, stimulation parameters, patient selection criteria, and outcome assessment methods. To account for this clinical and methodological variability, a random-effects model was employed in our meta-analysis. This approach provides a more conservative and robust estimate of treatment effect in the presence of heterogeneity, thereby strengthening the validity of the overall findings despite inter-study differences.

Three additional studies were evaluated but not included in the meta-analysis. Lin et al. reported a case of CP with severe dystonia in a 21-year-old female due to hypoxia at birth [42]. The patient did not benefit from GPi DBS surgery and was subsequently treated with high-frequency DBS on superior cerebellar peduncles (SCPs). After 6 months of follow-up, there was a reported improvement of 36.4% in the BFMDRS-M score (pre-operation = 93.5, post-operation = 59.5). Kim et al. conducted a study comparing bilateral GPi DBS with GPi DBS plus unilateral thalamotomy in 10 adult patients with CP [41]. The results did not show any significant improvement in the overall BFMDRS score between the two groups. Romina et al. reported a decrease in mean BFMDRS-M after 12 months of DBS implantation, but no change in individual patients [44]. Romina’s study employed a within-subject design comparing pre- and post-operative outcomes without including an external control group, making it incompatible with the meta-analysis criteria. Galanda et al. reported positive improvements in posture and spasticity in CP patients through anterior cerebellar stimulation [45].

## Factors influencing the efficacy of DBS

While patients with dystonia due to CP may potentially benefit from DBS, it is generally believed that the response to DBS treatment in CP patients with dystonia is not as favorable as in patients with idiopathic or genetic dystonia. This observation is particularly noteworthy considering the greater variability associated with acquired dystonia compared to its inherited or idiopathic counterparts [38, 46, 47]. The poorer response in patients with acquired dystonia is thought to be attributed to brain network damage, which limits the effectiveness of neuromodulation [48]. Therefore, there is a critical clinical need to identify prognosis and clinical predictors of surgical intervention for dystonia patients undergoing DBS in order to improve treatment outcomes.

### Demographic criteria

In a recent meta-analysis, it was found that disease duration and age at surgery were weakly negatively associated with percent improvements in BFMDRS scores, as measured by the Pearson correlation coefficient. Through stepwise multiple regression analysis, only disease duration was identified as a significant predictor (β = − 0.165, P = 0.006). This finding highlights the positive correlation between shorter disease duration and greater efficacy of DBS treatment. On the other hand, factors such as age at onset, follow-up period, and BFMDRS motor and disability scores at baseline were found to have no association with the clinical outcomes [49]. In contrast, Romina et al. suggested that the age of dystonia onset was related to DBS response, with less favorable results in early-onset forms [44]. Additionally, Isaias et al. found that disease duration and age at operation exhibited a significant negative correlation with DBS outcomes [50].

### Structural brain lesions

The depiction of the brain on the preoperative magnetic resonance imaging (MRI) scan was an essential predictor for a favorable surgical response [47]. Researchers found a significant difference in Global Outcome Scale (GOS) scores between patients with normal and abnormal MRI scans, indicating that those with normal scans had a better prognosis. Elkaim et al. also emphasized that the presence of underlying brain pathology—whether inherited or acquired—is associated with less favorable DBS outcomes in pediatric patients. In their analysis, brain pathologies such as pantothenate kinase-associated neurodegeneration, Lesch-Nyhan disease, and choreiform athetosis were discussed as examples of conditions linked to reduced therapeutic efficacy [51].

### Target selection for DBS

The choice of target and the success of DBS treatment depend on the precise diagnosis, individual’s condition, and expertise of the neurosurgical team. When selecting targets for DBS surgery to treat dystonia, the most commonly used nucleus is GPi. This nucleus is generally applicable to both primary and secondary dystonia [52]. As the principal output structure of the basal ganglia, the GPi primarily projects to the thalamus and midbrain, allowing it to control contralateral limb movement [53]. However, regulating all parts of the GPi and associated globus pallidus circuits can be challenging due to the large size and potential structural abnormalities of the GPi. This is particularly true for patients with perinatal injuries, such as those with CP [41]. As a result, alternative targets such as STN, ventral intermediate nucleus (Vim), ventralis oralis anterior (Voa), ventralis oralis posterior (Vop), and PPN have been proposed for CP patients with severe dystonia [31, 40, 54].

In a recent meta-analysis, patients who underwent GPi-DBS and STN-DBS showed significant improvements in movement symptoms, disability symptoms, 36-item Short Form Health Survey (SF-36) scores, and Beck Depression Inventory (BDI) scores [55]. Although the mean movement and disability scores were slightly higher in the STN group compared to the GPi group, the difference was not statistically significant. STN-DBS helps regulate abnormal activity patterns in the basal ganglia, leading to improved motor control. The STN is located upstream of the GPi in the motor circuit of the basal ganglia, suggesting that stimulation of the STN may be more effective than stimulation of GPi [52]. Schjerling et al. found that BFMDRS-M scores improved by 13.8 points with STN stimulation and 9.1 points with GPi stimulation after 6 months of follow-up (p = 0.08) [56]. Lin et al. also reported a significantly larger percentage improvement in the BFMDRS total movement score after 1 month of follow-up with STN-DBS (64%) compared to GPi-DBS (48%) (p = 0.01) [57]. Although the STN has shown promise as a potential target for DBS in certain movement disorders, its small size and deep anatomical location make accurate localization more challenging, especially in pediatric patients. Additionally, the STN has been less extensively investigated in this population, whereas the GPi remains the most well-established and commonly used target with a more robust evidence base supporting its efficacy.

The Voa and Vop serve as the principal outputs of the globus pallidus, receiving retroactive feedback from the motor cortex. Thalamic DBS intervenes in the tremor circuitry, reducing or eliminating tremors. In individuals with dystonia, the neuronal firing of the ventralis oralis occurs at the same frequency as the STN and GPi. These aberrant neuronal activities can be inhibited or modulated by DBS or lesioning [58]. Katsakiori et al. documented a favorable functional outcome in a patient with secondary dystonia who underwent Voa-DBS [27]. Additionally, Ghika et al. reported significant improvement in a patient with dystonia after systemic hypoxia who was treated with GPi and Voa stimulation, specifically after Voa-DBS surgery [59]. Previous studies have shown that around 83% of dystonic-related activity is located in the Vim region, which is likely associated with the upper limb [58]. Kim et al. demonstrated that patients who underwent GPi plus Vim DBS had significantly improved movement in the contralateral upper limb and better health-related quality of life compared to patients who underwent GPi surgery alone [31]. Dual-target DBS, particularly combining the thalamus with either the GPi or subthalamic nucleus, has shown superior efficacy in managing coexisting dystonia and tremor compared to single-target approaches. Previous study reported that Vim DBS provided an 85% tremor reduction but limited dystonia relief, while GPi DBS achieved 64% improvement in dystonia with only 40% tremor reduction [60]. Several patients required subsequent implantation of a second target (Vim or GPi) to manage residual symptoms, suggesting a complementary effect of these targets. Objective motion assessments further supported greater tremor control with combined stimulation compared to either target alone. These findings, though based on limited case series, highlight the potential of dual-target DBS to optimize outcomes in patients with complex movement disorders involving both tremor and dystonia.

The cerebellum has shown promise as a potential therapeutic target for individuals with CP. According to Dr. Ross Davis, 600 patients with CP have received cerebellar stimulation treatment, resulting in improvement for about two-thirds to three-quarters of the patients. Additionally, at least 50% of the patients experienced a reduction in spasticity of more than 20% [61]. A case study conducted by Stroud et al. also demonstrated significant benefits from cerebellar cortical stimulation, leading to a more than 50% decrease in BFMDRS-M scores for a patient with acquired dystonia [62]. Another study has reported four cases of cerebellar DBS with functional improvement that might be useful in the specific population of patients with dystonia [45]. Furthermore, DBS targeting the PPN shows promise in addressing locomotion and balance issues, potentially improving gait-related problems [54].

### Stimulation programming

Stimulation programming in patients with CP showed significant heterogeneity. While there is no consensus on the optimal stimulation settings, several advanced programming approaches can potentially maximize clinical benefit [63]. A recent meta-analysis provided the average stimulation parameters for STN-DBS for acquired dystonia, including an amplitude of 2.56 ± 0.73 V, pulse width of 100.61 ± 40.62 μs, and frequency of 143.79 ± 34.87 Hz [64]. Additionally, Magown et al. reported prevalent stimulation parameters for CP patients, with an amplitude of 3.0 V, frequency of 130 Hz, and pulse width of 120 μs. [65]. Moro et al. emphasized that frequency and amplitude were crucial factors in determining the clinical benefit of GPi-DBS [66]. In addition, the marginal effects of interleaving stimulation (a programming strategy that alternates stimulation between two sets of contacts to optimize therapeutic benefit) in reducing adverse effects and enhancing clinical outcomes were evident [67].

### Follow-up and long-term care

Regular follow-up appointments with the healthcare team are important for monitoring progress, making any necessary adjustments to the stimulation parameters, and addressing any issues that may arise [68]. The overall success of DBS interventions is significantly influenced by the patient’s psychological and emotional well-being, as well as the support system. Research studies have indicated that music, when used as a complementary form of therapy, can assist individuals with CP in overcoming emotional challenges and reducing levels of anxiety and pain [69]. After 30 min of intervention, the modified Yale preoperative anxiety scale for children’s anxiety score of the music group was significantly lower than that of the control group (t = 4.72, P = 0.00).

## Measures to improve the efficacy of DBS

### Comprehensive patient assessment and appropriate patient selection

A thorough assessment is essential in confirming the diagnosis of dystonia and evaluating the severity, type, and distribution of symptoms. Motor dystonia is characterized by abnormal movements, often associated with dysfunction in the basal ganglia and cerebral cortex, which can result in difficulties initiating and controlling voluntary movements. On the other hand, postural dystonia primarily involves maintaining abnormal bodily positions, possibly due to irregular muscle tone and disrupted sensory feedback mechanisms, with potential involvement from the cerebellum. It is crucial to determine the specific type of dystonia exhibited by the patient. Candidates eligible for surgical intervention are those who have not responded adequately to other treatments, are medically suitable for surgery, and have realistic expectations regarding potential outcomes.

### Advancements in imaging modalities

Most patients with dystonia do not have significant lesions on routine clinical imaging. However, the application of comprehensive imaging techniques such as positron emission tomography and MRI have revealed subtle quantitative abnormalities that are crucial for ensuring precise and safe electrode implantation [70]. With advancements in imaging modalities, diffusion tensor imaging (DTI) has become a valuable tool for visualizing white matter tracts formed by axonal projections of cortical and subcortical neurons *in vivo*. This allows for better optimization of DBS electrodes and stimulation paradigms, focusing on tract stimulation rather than deep brain nuclei introduction [71]. The White Matter Attenuated Inversion Recovery (WAIR) sequence is particularly useful in targeting the normally MRI-indistinguishable VIM, providing accurate preoperative planning, direct targeting, and anatomical analysis for DBS surgery [72]. Additionally, it is important to ensure that the electrodes reach the optimal depth for effective response to stimulation. Deviating more than 2 mm from the intended target can significantly impair treatment effectiveness or require higher stimulus energy, leading to intolerable adverse reactions [63]. Therefore, obtaining high-quality preoperative and intraoperative MRI images is crucial for optimizing the efficacy of DBS procedures.

### Optimal target selection for DBS intervention

The determination of the optimal target for deep brain stimulation (DBS) for dystonia is dependent on a comprehensive assessment of individual cases. This assessment considers factors such as the specific type and distribution of dystonia, previous responses to medications and other treatments, unique patient attributes, and surgical expertise. The GPi is the primary target for dystonia, as it is involved in both motor and postural dystonia through basal ganglia pathways. By modulating activity in this region, DBS can improve motor control and reduce abnormal movements. STN is less frequently used for dystonia. However, existing literature suggests that it may be more effective in certain specific circumstances and should be considered accordingly. In cases of postural dystonia, especially when tremor is a significant component of the condition, targeting the thalamus, specifically the Vim, may be a relevant consideration.

### Pharmacological administration

Besides DBS surgery to treat dystonia in patients with CP, there are various pharmacological management options that can be used in conjunction with surgical therapy. These options aim to preserve or restore function, relieve abnormal posture, and minimize pain [73]. Despite Multiple medications have been evaluated, including oral baclofen, benzodiazepines, clonidine, gabapentin, levodopa, benzhexol, botulinum toxin, and intrathecal baclofen (ITB), the availability of high-quality evidence remains limited [74]. Currently, existing evidence does not strongly support the use of oral medications or botulinum toxin alone to effectively reduce dystonia in individuals with CP, likely due to the complex and multifactorial nature of dystonic pathophysiology. Among neurosurgical alternatives, ITB has shown promise, particularly in reducing spasticity and associated symptoms. ITB may be considered a secondary option for patients who do not experience adequate symptom control following DBS. However, due to the considerable heterogeneity in treatment response among patients with CP, these conclusions should be interpreted with caution. Further well-designed clinical trials are needed to clarify the optimal therapeutic approaches for this population.

### Long-term DBS management

The programming of DBS can begin immediately after confirming the correct position of the DBS lead and ensuring there are no complications from the procedure. Alternatively, it can be initiated several weeks after surgery to address potential improvements related to the effects of lead insertion [75]. Despite the lack of consensus on the best stimulation parameters, it is highly recommended to take a systematic approach to optimize these settings [76]. Fine-tuning the parameters based on patient feedback and clinical assessment is an ongoing process. Real-time recording of neurophysiological biomarkers based on the patient’s clinical status can be used to optimize stimulation parameters according to individual needs [77]. Specialized visualization software can estimate the volume of activated tissue and assist in programming by visualizing activation maps based on pre-defined target maps [78]. The response to DBS in patients with CP varies greatly, and dystonic symptoms often continue to progress and fluctuate during the follow-up period after DBS. Alongside the long-lasting effectiveness of DBS in treating dystonia, there have been cases of worsening symptoms and tolerance to treatment, necessitating staged or rescue DBS interventions. Post-surgery, intensive rehabilitation and physical therapy should be provided to help patients maximize their improved motor abilities. In the field of nursing, the phenomenological interview and theoretical tools of phenomenology are used to gain a deeper understanding of the lives of CP patients [79]. Caregivers should approach these interviews with an open and empathetic attitude, aiming to establish a foundation of trust with the interviewee. It is crucial for the caregiver to actively engage with the interviewee, encouraging them to provide detailed descriptions that are relevant to their experiences. The provision of comprehensive care, led by a multidisciplinary team including neurologists, neurosurgeons, neuropsychiatrists, nurses, and rehabilitation specialists, is crucial for delivering holistic care and achieving optimal outcomes. Therefore, the long-term management of DBS is a dynamic process that requires collaborative efforts between healthcare professionals, patients, and their caregivers.

## Conclusion

DBS has proven to be an effective therapeutic intervention for dystonia in selected patients with CP. However, the clinical response to DBS can vary significantly depending on several factors, including disease duration, the presence of structural brain abnormalities, target site selection, stimulation programming, and the quality of long-term multidisciplinary care. Optimizing DBS outcomes requires continuous advancements in neuroimaging technologies, thorough patient selection and evaluation, tailored pharmacological management, and close collaboration between patients, caregivers, and healthcare professionals. A personalized, patient-centered approach—alongside regular assessment and adjustment—is essential to achieving the best possible clinical results. Promising innovations such as brain sensing, closed-loop DBS, and remote programming offer significant potential to further improve motor symptoms and enhance quality of life in CP patients. Nevertheless, to fully elucidate the therapeutic role of DBS in dystonic CP, further research is warranted. In particular, prospective cohort studies with large sample sizes conducted under standardized protocols and using a multidimensional assessment framework are needed to systematically evaluate the impact of DBS on both motor function and quality of life.
