# Supplementary material for: Deep brain stimulation for dystonia treatment in cerebral palsy: efficacy exploration
Source: Exp Biol Med (Maywood). 2025 Jun 9;250:10330. doi: 10.3389/ebm.2025.10330 (PMC12183516; doi:10.3389/ebm.2025.10330)
Supplement: Supplementary file 1 [file DataSheet1.docx]

**Eligibility and** **exclusion criteria**

**Inclusion Criteria:**

1. Diagnosis of Cerebral Palsy (CP): Patients must have a confirmed diagnosis of cerebral palsy, which may be established through clinical evaluation and/or neuroimaging.
2. Presence of Dystonia: Patients should exhibit significant dystonic symptoms that significantly impact their daily functioning and quality of life.
3. Failure of Conservative Treatment: Patients must have undergone and failed to adequately respond to conservative treatments for dystonia, which may include physical therapy, oral medications, and other non-invasive interventions.
4. Cognitive and Physical Suitability: Patients should be cognitively and physically capable of undergoing deep brain stimulation (DBS) surgery and subsequent follow-up care.
5. Willingness and Informed Consent: Patients and/or their legal guardians should provide informed consent for the DBS procedure, including a thorough understanding of potential risks, benefits, and alternative treatments.

**Exclusion Criteria:**

1. Medical Instability: Patients with unstable medical conditions that pose a high surgical risk may be excluded. This includes uncontrolled systemic illnesses or severe co-morbidities.
2. Active Infections: Patients with active infections, especially in the central nervous system, may be excluded to minimize the risk of post-operative complications.
3. Psychiatric Disorders: Severe untreated psychiatric conditions or uncontrolled behavioral issues that may interfere with post-operative care and device management.
4. Contraindications for Surgery: Any contraindications for undergoing neurosurgical procedures, such as intracranial abnormalities, may exclude a patient from DBS.
5. Unrealistic Expectations: Patients or caregivers with unrealistic expectations regarding the outcomes of DBS, or those unable to participate in post-operative programming and rehabilitation, may be excluded.
6. Inability to Comply with Follow-up: Patients who are unlikely or unable to comply with the necessary follow-up appointments and adjustments to the DBS system.

**Data analysis**

We used Mean Differences (MDs) for continuous variables measured using the same instrument. The heterogeneity test was carried out by I² test. The results showed low heterogeneity (I² < 50); therefore, the fixed-effect models were used for the analyses. RevMan 5.4 statistical software provided by Cochrane collaboration Network was used for statistical analysis. The continuous variable used mean ± standard deviation as the effect index, and the interval estimation of weighted mean difference (WMD), effect value was expressed by 95% confidence interval (CI). The test level of the effect was α = 0.05.
